# Treatment for Ulceration of Stomach and Cancreum Oris

**Published:** 1871-01

**Authors:** 


Treatment for Ulceration of Stomach and Cancreum Oris.—A mild aperient of rhubarb and magnesia. Wash the mouth every few hours with a weak solution of Chloride of Soda, and give five grains of Chlorate of Potass., dissolved in a little sweetened water every four hours. In some cases, alteratives and tonics may be added.

